# Inhibition of NRF2 by PIK-75 augments sensitivity of pancreatic cancer cells to gemcitabine

**DOI:** 10.3892/ijo.2013.2229

**Published:** 2013-12-23

**Authors:** HONG-QUAN DUONG, YONG WEON YI, HYO JIN KANG, YOUNG BIN HONG, WENXI TANG, ANTAI WANG, YEON-SUN SEONG, INSOO BAE

**Affiliations:** 1 Departments of Oncology, Georgetown University, Washington, DC, USA; 2 Radiation Medicine, Lombardi Comprehensive Cancer Center, Georgetown University, Washington, DC, USA;; 3 Department of Nanobiomedical Science and WCU (World Class University) Research Center of Nanobiomedical Science, Dankook University, Cheonan, Republic of Korea;; 4 Department of Biostatistics, Columbia University, New York, NY, USA; 5 Herbert Irving Comprehensive Cancer Center, Columbia University, New York, NY, USA

**Keywords:** PIK-75, gemcitabine, NRF2, MRP5, synergism

## Abstract

We describe the potential benefit of PIK-75 in combination of gemcitabine to treat pancreatic cancer in a preclinical mouse model. The effect of PIK-75 on the level and activity of NRF2 was characterized using various assays including reporter gene, quantitative PCR, DNA-binding and western blot analyses. Additionally, the combinatorial effect of PIK-75 and gemcitabine was evaluated in human pancreatic cancer cell lines and a xenograft model. PIK-75 reduced NRF2 protein levels and activity to regulate its target gene expression through proteasome-mediated degradation of NRF2 in human pancreatic cancer cell lines. PIK-75 also reduced the gemcitabine-induced NRF2 levels and the expression of its downstream target MRP5. Co-treatment of PIK-75 augmented the antitumor effect of gemcitabine both *in vitro* and *in vivo*. Our present study provides a strong mechanistic rationale to evaluate NRF2 targeting agents in combination with gemcitabine to treat pancreatic cancers.

## Introduction

Pancreatic cancer is one of the deadliest human cancer types with an estimated 45,220 new cases and 38,460 deaths in 2013 in US (1). Although pancreatic cancer accounts for ∼7% of all cancer deaths and ranks 4th as a cause of cancer death, the incidence and mortality rates increased for the overall US during 2000–2009 (1). In addition, the five-year survival rate is estimated as <5–6% (2). Around 90% of all pancreatic cancers are adenocarcinomas that originate in the epithelial cells of the pancreatic duct (3,4). Since the early stage pancreatic cancer usually has no detectable symptoms, only ∼15 to 20% of pancreatic cancer cases are diagnosed early enough to be eligible for surgery that provides the only chance of cure for pancreatic cancer patients (1). Gemcitabine, the recommended first-line chemotherapeutics, can be given alone or in combination with other drugs (1,5–7); however chemotherapy of pancreatic cancers is limited by innate or acquired resistance (8).

The nuclear factor erythroid 2-related factor 2 (NRF2) is a master regulator of genes involved in oxidative stress response, detoxification and drug resistance (9–12). As a member of basic region leucine zipper transcription factors, NRF2 binds to a DNA sequence called antioxidant response element (ARE) and induces transcription of target genes (9–12). Under normal reducing conditions, NRF2 is repressed by Kelch-like erythroid cell-derived protein with CNC homology-associated protein 1 (KEAP1)-dependent ubiquitination-proteasomal degradation (9–12). In addition, NRF2 is also downregulated by CR6-interacting factor 1 (CRIF1) under both reducing and oxidative stress conditions (13). While NRF2 decreases tumor susceptibility in most carcinogenesis models, constitutive activation of NRF2 may enhance tumor cell proliferation and/or protection against chemotherapy (10–12,14). In fact, NRF2 is upregulated in many types of tumors through somatic mutations that disrupt NRF2-KEAP1 regulation (10–12,15). More recently, it has been reported that several oncogenes, including K-Ras, B-Raf and Myc, increased the transcription of Nrf2 gene in primary murine cells to activate antioxidant and detoxification programs preferable for oncogenesis (16). Genetic targeting of K-Ras^G12D^-driven Nrf2 impaired *in vivo* tumorigenesis (16). Silencing NRF2 by RNA interference also inhibited tumor growth and increased efficacy of chemotherapy (17) or EGF-driven proliferation (18) in non-small cell lung cancer models and reduced the proliferation and drug-resistance in human lung cancer cells (19) or human pancreatic cancer cells (20,21). Taken together, NRF2 pathway is a plausible therapeutic target for cancer therapy.

In this study, we identified PIK-75 as an agent to down-regulate NRF2 protein level and demonstrated its application in combination with gemcitabine to further reduce *in vivo* tumor growth of human pancreatic cancer.

## Materials and methods

### Cell culture and reagents

MIA PaCa-2 cells were purchased from American Type Culture Collection (Manassas, VA, USA) and AsPC-1 cells were obtained from Tissue Culture Shared Resource of Georgetown University Medical School. MIA PaCa-2 cells were maintained in Dulbecco’s modified Eagle’s medium (DMEM) containing 10% heat-inactivated fetal bovine serum (HI-FBS; HyClone, Logan, UT, USA), 2.5% horse serum (HS) and 100 U/ml penicillin/streptomycin. AsPC-1 cells were cultured in RPMI-1640 media supplemented with 20% HI-FBS, 100 U/ml penicillin/streptomycin and 1 mM sodium pyruvate. Cell culture reagents were purchased from BioWhittaker (Walkersville, MD, USA), Lonza (Basel, Switzerland), Invitrogen (Carlsbad, CA, USA) or Cellgro (Manassas, VA, USA). Viable cells were monitored by the Luna Automated Cell Counter (Logos Biosystems, Gyunggi-do, Korea). Small molecule compounds were purchased from the following sources: PIK-75, PI-103, brivanib, TAE-684, XL-880, enzastaurin, GDC-0879, deforolimus and TGX221 from Selleck Chemicals (Houston, TX, USA); BMS-754807 from MedKoo (Chapel Hill, NC, USA); dasatinib, everolimus and ZSTK474 from LC Labs (Woburn, MA, USA); and tertbutylhydroquinone (tBHQ) and MG132 from Sigma (St. Louis, MO, USA). Compounds were dissolved in dimethyl sulfoxide (DMSO) and stored at −20°C in small aliquots. Gemcitabine was obtained from Sigma and dissolved in phosphate-buffered saline (PBS).

### Cell proliferation assay

Cells in 6-well plates were transfected with 100 nM of either control- or NRF2-siRNA (20) by Lipofectamine 2000 reagent (Invitrogen). Four hours after transfection, equal volume of fresh media were added to each well. The cells were trypsinized and the number of viable cells was counted by trypan blue dye exclusion assay every day. After counting, the cell lysates from harvested cells were subjected to western blot analysis.

### 3-(4,5-Dimethylthiazol-2-yl)-2,5-diphenyltetrazolium bromide (MTT) assay

A total of 2,000 human pancreatic cancer cells (MIA PaCa-2 or AsPC-1) per well were plated in 96-well flat-bottom plates and then treated with either gemcitabine, PIK-75 alone or in combination of both drugs with indicated concentrations. At the indicated times, 20 *μ*l of 1 mg/ml MTT (Sigma) in PBS was added to each well and further incubated for ∼4 h. After centrifugation and removal of the medium, 150 *μ*l of DMSO (Sigma) was added to each well to dissolve the formazan crystals. The absorbance was measured at 562 nm using an ELx808 absorbance microplate reader (BioTek Instruments, Inc., Winooski, VT, USA). Absorbance of untreated cells was designated as 100%, and the relative viable cells were expressed as a percentage of this value. The drug interaction was evaluated by using the combination index (CI) according to the method of Chou and Talalay (22) using CompuSyn software (ComboSyn, Inc., Paramus, NJ, USA).

### Transfection and reporter gene assay

Cell culture, seeding and DNA plasmid transfection were performed as previously reported (23,24). The luciferase activity was measured according to manufacturer’s instruction (Promega, Madison, WI, USA) using Victor2 plate reader (Perkin-Elmer, Waltham, MA, USA) at the Genomics and Epigenomics Shared Resource of Georgetown University Medical Center and normalized to β-galactosidase activities.

### Clonogenic assay

MIA PaCa-2 or AsPC-1 cells (2×10^5^ cells) were seeded in 60-mm dishes. Twenty-four hours after plating, various concentrations of PIK-75 were added to each dish. After treatment for 24 h, cells were trypsinized and re-seeded in 60-mm dishes at a density of 500 cells per dish in triplicate. The cells were further incubated for 14 days and stained with 0.5% crystal violet in PBS containing 25% methanol. Colonies were examined under a light microscope and counted after capturing images by scanner. Relative colony numbers were calculated as a percentage of the untreated cells (25).

### Western blot analysis

MIA PaCa-2 or AsPC-1 cells were grown to ∼70% confluency and treated with drugs as indicated. Cells were lysed by lysis buffer containing 20 mM Tris-HCl, 0.5 M NaCl, 0.25% Triton X-100, 1 mM EDTA, 1 mM EGTA, 10 mM β-glycerophosphate, 10 mM NaF, 300 *μ*M Na_3_VO_4_, 1 mM benzamidine, 2 *μ*M PMSF and 1 mM DTT. The protein concentration was determined by the BCA protein assay (Thermo Scientific, Rockford, IL, USA). Proteins were separated on SDS-PAGE, transferred on to PVDF membrane, blocked in 1X blocking buffer (Sigma) and probed with the following antibodies: phospho-AKT (S473), phospho-GSK3β (S9), AKT, GSK3β, X-linked Inhibitor of Apoptosis Protein (XIAP), and Poly-ADP-Ribose-Polymerase (PARP) from Cell Signaling Technology (Boston, MA, USA); NRF2, multi-drug resistance associated protein 5 (MRP5), and survivin from Santa Cruz Biotechnology (Santa Cruz, CA, USA); Glutamate-Cysteine Ligase Catalytic subunit (GCLC) from Novus Biologicals (Littleton, CO, USA); Heme Oxygenase-1 (HO-1) from Stressgen Biotechnologies (Victoria, BC, Canada); NAD(P)H:quinone oxidoreductase 1 (NQO1) and GFP from Abcam (Cambridge, MA, USA); and FLAG (M2) and β-actin from Sigma. Then, the membranes were incubated with horseradish peroxidase (HRP)-conjugated secondary antibodies (Sigma), incubated with a chemiluminescence reagent (Santa Cruz Biotechnology) according to the manufacturer’s recommendation, and exposed to X-ray film (American X-ray and Medical Supply, Jackson, CA, USA).

### DNA binding assay

AsPC-1 cells treated with various concentrations of PIK-75 for 24 h were used to prepare nuclear extracts. The DNA binding activity was determined by TransAM NRF2 assay kit (Active Motif, Carlsbad, CA, USA) according to the manufacturer’s protocol.

### Caspase-3/7 activity assay

MIA PaCa-2 or AsPC-1 cells were treated as indicated and caspase-3/7 activity was measured from cell lysates with Caspase-3/7 Glo Assay kit (Promega) according to the manufacturer’s protocol. Luminescence was measured using Victor X or Victor^2^ multilable plate readers (Perkin-Elmer Life Sciences, Boston, MA, USA). Relative luminescence units were determined by calculating luminescence values from samples as a percentage of values from vehicle-treated control samples. The experiments were performed in triplicate and repeated on two separately initiated cultures.

### Reverse transcriptase (RT)-PCR and quantitative real-time PCR (qRT-PCR)

RT-PCR and qRT-PCR were performed as described previously (23) with an Applied Biosystems-Prism Sequence Detector System 7900HT at the Genomics and Epigenomics Shared Resource of Georgetown University Medical Center. The following primer were used: NRF2 forward, 5′-aaa cca ccc tga aac gac ag-3′ and reverse, 5′-agc ggc ttg aat gtt tgt c-3′; GCLC forward, 5′-ctg ggg agt gat ttc tgc at-3′ and reverse, 5′-agg agg ggg ctt aaa tct ca-3′; HO-1 forward, 5′-agg tca tcc cct aca cac ca-3′ and reverse, 5′- tgt tgg gga agg tga aga ag-3′; MRP5 forward, 5′-acc cgt tgt tgc cat ctt ag-3′ and reverse, 5′-tct gtc aac agc cac tga gg-3′; β-actin forward, 5′-gct atc cct gta cgc ctc tg-3′ and reverse, 5′-ata tct gct gga agg tgg ac-3′; GAPDH forward, 5′-gta tga caa cga att tgg cta cag -3′ and reverse, 5′-agc aca ggg tac ttt att gat ggt-3′.

### Tumor xenograft study

Animal use procedures were approved by the Institutional Animal Care and Use Committee of Georgetown University Medical Center. MIA PaCa-2 cells (∼1.7×10^6^ cells/mouse) mixed with Matrigel (BD Biosciences, San Jose, CA, USA) were injected subcutaneously into the flank of male athymic nude (Foxn1nu) mice aged 6-weeks (Harlan Laboratories, Frederick, MD, USA). Gemcitabine (50 mg/ml) was dissolved in PBS and PIK-75 (20 mg/ml) was dissolved in DMSO. Injection solution was made as 10% of Cremophor^®^ EL (Sigma) and 3% of poly(ethylene glycol) 400 (Sigma) in sterile water. Before administration of compounds, gemcitabine was further diluted in PBS and DMSO or PIK-75 was further diluted in the injection solution and sterilized by 0.2 *μ*m filter unit. These diluents were mixed with 1:1 ratio and administered into peritoneal cavity of the mouse. Gemcitabine (20 mg/kg) or gemcitabine (20 mg/kg)/PIK-75 (2 mg/kg) combination was administered twice per week and vehicle control and PIK-75 (2 mg/kg) were administered 5 times per week. The body weights and tumor sizes were measured 3 times per week. Tumor volumes were calculated as width (mm) × length (mm) × height (mm)/2.

### Statistical analysis

For multiple comparisons, analysis of variance (ANOVA) using Tukey’s multiple comparison adjustments and subsequent two-sample t-tests were conducted for comparison. All statistical tests were two-tailed and employed at a significance level of 5% to determine whether a significant difference exists in the assigned experiments. Data were analyzed using SAS version 9.3. ^*^P≤0.05; ^**^P≤0.01; and ^***^P≤0.001.

## Results

### NRF2 is essential for the proliferation of pancreatic cancer AsPC-1 cells

**Figure 1.**
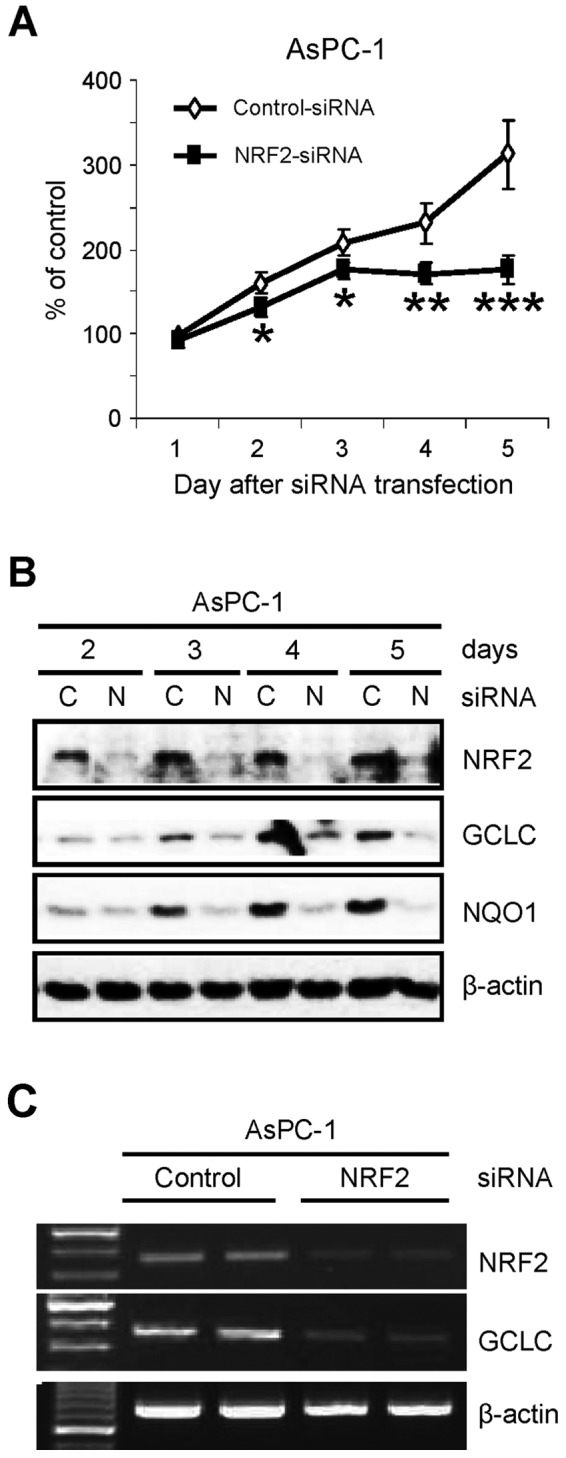
NRF2-knockdown reduces the proliferation of pancreatic cancer AsPC-1 cells. (A) AsPC-1 cells were transfected with siRNA (NRF2 vs. control) and the viable cells were determined by trypan blue assay over the time. Data are presented as mean ± SEM from three independent experiments performed in duplicate. ^*^P≤0.05; ^**^P≤0.01; and ^***^P≤0.001. (B) The cell lysates from experiment described in (A) were analyzed by western blot analysis with indicated antibodies. β-actin was used as a loading control. (C) The cDNAs made from total RNAs of cells transfected with siRNA were analyzed by RT-PCR analysis with primers for indicated genes.

Previously, we found that NRF2 protein is abnormally elevated in pancreatic cancer tissues and cell lines (20). To further investigate the role of NRF2 in pancreatic cancer cells, we determined the proliferation of pancreatic cancer cells after knockdown of NRF2. After transfection of siRNA, the number of viable cells was determined by trypan blue dye exclusion assay. Similar to recent reports in lung cancer cells (17–19), knockdown of NRF2 (NRF2-KD) by siRNA reduced the proliferation of AsPC-1 pancreatic cancer cells over the times tested (Fig. 1A). As confirmation, the cell lysates from the same experiment were analyzed by western blot analysis. NRF2-KD was evident on the day after siRNA transfection and maintained up to 5 days after transfection (Fig. 1B). As expected, NRF2-KD reduced the protein level of its downstream genes such as GCLC and NQO1 (Fig. 1B). Suppression of NRF2-dependent transcription in NRF2-KD cells was further analyzed by RT-PCR analysis. The reduction of GCLC protein (Fig. 1B) was well correlated with the reduction of its mRNA in NRF2-KD cells (Fig. 1C).

### Identification of PIK-75 as an inhibitor of NRF2-dependent transcription

**Figure 2.**
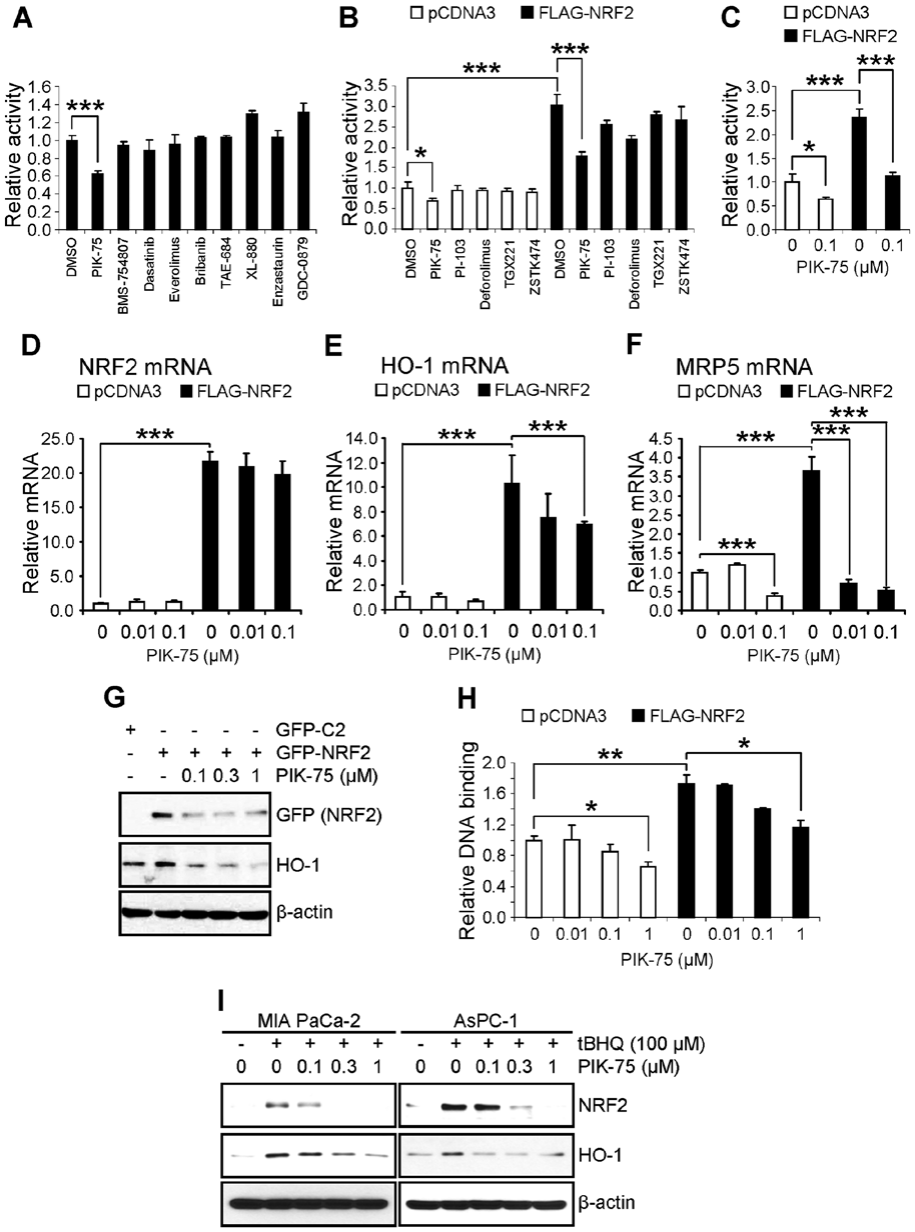
PIK-75 reduces NRF2 transcriptional activity in pancreatic cancer cells. (A) AsPC-1 cells, transfected with the NRF2 reporter gene ARE-Luc, were further treated with 0.1 *μ*M of various protein kinase inhibitors for 24 h and the reporter activities were determined. (B) AsPC-1 cells were transfected with ARE-Luc and pCDNA3 or FLAG-NRF2. The ARE-Luc reporter gene activity was determined from the cells treated with 0.1 *μ*M of various PI3K/AKT/mTOR inhibitors for 20 h. (C) MIA PaCa-2 cells were transfected with ARE-Luc and pCDNA3 or FLAG-NRF2. The cells were further treated with PIK-75 for 8 h and luciferase activity was determined. (D) The levels of NRF2 mRNAs were determined by qRT-PCR in the AsPC-1 cells transfected with pCDNA3 or FLAG-NRF2 followed by treatment of PIK-75 for 24 h. (E) The levels of HO-1, a target gene of NRF2, were determined by qRT-PCR in the same samples used in (D). (F) The levels of MRP5 mRNA were determined by qRT-PCR in the same samples used in (D). (G) AsPC-1 cells, transfected with either pGFP-C2 or GFP-NRF2, were treated with increasing amount of PIK-75 for 8 h and the levels of proteins were analyzed by indicated antibodies. (H) DNA binding activity of NRF2 was determined in the nuclear extracts from the AsPC-1 cells transfected with pCDNA3 or FLAG-NRF2 and further treated with different concentrations of PIK-75 for 24 h. (I) The MIA PaCa-2 and AsPC-1 cells, treated with 100 *μ*M of tBHQ for 1 h, were further treated with increasing concentration of PIK-75 for 8 h. Western blot analysis was performed with indicated antibodies. (A–F and H) Data are presented as mean ± SD from a representative experiment performed in triplicate. ^*^P≤0.05; ^**^P≤0.01; and ^***^P≤0.001. (G and I) β-actin was used as a loading control.

NRF2 is tightly regulated by proteasomal degradation that mediated by KEAP1 (9–12) or CRIF1 (13). Since NRF2 can be activated by post-translational modification such as phosphorylation by various protein kinases (11), it is plausible that activation of NRF2 is mediated by oncogenic activation of upstream signaling pathway in cancer cells. We postulated that NRF2 could be downregulated by small molecule kinase inhibitors targeting the proper signaling pathway. To identify the NRF2 downregulating inhibitor, AsPC-1 cells were transfected with a luciferase reporter gene construct containing the ARE from NQO1 gene (ARE-Luc) and treated with various kinase inhibitors (0.1 *μ*M) for 24 h. Interestingly, PIK-75, known as a PI3K/DNA-PK inhibitor (26), significantly reduced the ARE-Luc activity in AsPC-1 cells (Fig. 2A). We further compared a series of inhibitors (0.1 *μ*M) targeting PI3K/AKT/mTOR pathway in the AsPC-1 cells transfected with FLAG-NRF2 and ARE-Luc. Kinase inhibitors targeting this pathway reduced the ARE-Luc reporter activity in the presence of FLAG-NRF2 to certain degree, but PIK-75 was the most potent inhibitor that reduced the ARE-Luc reporter expression upto ∼50% either in the absence or presence of exogenous FLAG-NRF2 (Fig. 2B). The PIK-75-mediated suppression of NRF2 transactivation was further confirmed in another pancreatic cancer cell, MIA PaCa-2. The suppression of NRF2-mediated ARE-Luc activation by PIK-75 was as rapid as 8 h post-treatment in MIA PaCa-2 cells (Fig. 2C).

### PIK-75 inhibits NRF2-target gene expression by reducing NRF2 protein level

The effect of PIK-75 on the mRNA expression of NRF2 and its target gene was assessed by qRT-PCR in the cDNAs from AsPC-1 cells transfected with FLAG-NRF2 and treated with PIK-75. As expected, overexpression of FLAG-NRF2 (Fig. 2D) markedly increased the mRNA expression of NRF2 target gene HO-1 (Fig. 2E) and MRP5 (Fig. 2F). Under these conditions, PIK-75 reduced the NRF2-mediated expression of HO-1 and MRP5 mRNA (Fig. 2E and F). On the contrary, the effect of PIK-75 on the NRF2 mRNA was limited (Fig. 2D) and the NRF2 mRNA was slightly reduced at 1 *μ*M concentration of PIK-75 (data not shown).

The effect of PIK-75 on the level of NRF2 protein was also determined by western blot analysis. AsPC-1 cells were transfected with GFP-NRF2 and then treated with increasing amount of PIK-75 for 8 h. As results, the level of overexpressed GFP-NRF2 was decreased by PIK-75 in a dose-dependent manner (Fig. 2G). Consistent with mRNA expression, the level of HO-1 protein was also decreased by PIK-75 treatment under these conditions (Fig. 2G).

The effect of PIK-75 on the NRF2 activation was confirmed by NRF2-DNA binding activity. Nuclear extracts from AsPC-1 cells, transfected with FLAG-NRF2 and treated with PIK-75, were subject to the ELISA-based DNA binding assay. As shown in Fig. 2H, PIK-75 reduced the DNA-binding activity of the overexpressed FLAG-NRF2 as well as endogenous NRF2.

The effect of PIK-75 on the endogenous NRF2 was further determined by tBHQ activation model. Two pancreatic cancer cells, MIA PaCa-2 and AsPC-1 were activated by tBHQ for 1 h followed by PIK-75 treatment for 8 h. Again, PIK-75 reduced the levels of tBHQ-activated NRF2 and its downstream targets, HO-1 as early as 8 h post-treatment in both cells (Fig. 2I). Taken together, PIK-75 represses NRF2-target gene expression through downregulation of the NRF2 protein.

### PIK-75 induces proteasomal degradation of NRF2

**Figure 3.**
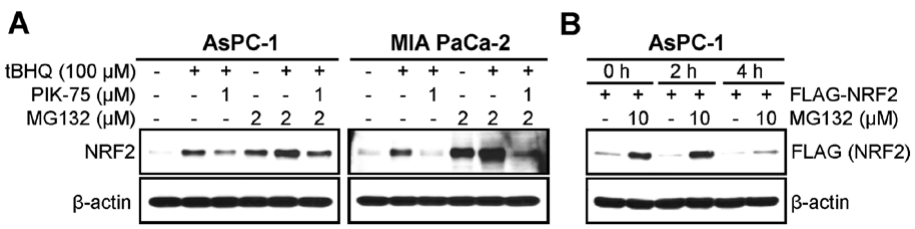
PIK-75 induces the proteasome-mediated degradation of NRF2. (A) AsPC-1 and MIA PaCa-2 cells, treated with 100 *μ*M of tBHQ for 1 h, were further treated with PIK-75 for 4 h in the absence or presence of MG132. (B) AsPC-1 cells, transfected with FLAG-NRF2, were further treated with 0.1 *μ*M of PIK-75 for indicated time in the absence or presence of MG132. (A and B) Western blot analysis was performed with indicated antibodies. β-actin was used as a loading control.

NRF2 is actively regulated by proteasomal degradation. Since PIK-75 reduced both endogenous and exogenous NRF2 protein, we further tested the PIK-75-mediated NRF2 downregulation in the presence of proteasome inhibitor. AsPC-1 cells were activated by tBHQ for 1 h followed by treatment of PIK-75 for 4 h in the absence or presence of the proteasome inhibitor MG132. Western blot analysis showed that treatment of MG132 alone induced the level of NRF2 protein similarly to that by tBHQ treatment (Fig. 3A left panel, lanes 2 vs. 4). This indicates that NRF2 is actively degraded by proteasome in this cell line. Indeed, co-treatment of tBHQ and MG132 further increased the NRF protein. Under this condition MG132 treatment was repressed the PIK-75-mediated reduction of NRF2 (Fig. 3A left panel, lanes 3 vs. 6). Inhibition of proteasome by MG132 also recovered by the PIK-75-mediated reduction of NRF2 in MIA PaCa-2 cells (Fig. 3A right panel).

AsPC-1 cells transfected with FLAG-NRF2 were briefly treated with PIK-75 in the absence or presence of MG132. Interestingly, it was evident that overexpressed FLAG-NRF2 was also actively regulated by proteasome (Fig. 3B, lanes 1 vs. 2). Under this experimental setting, the PIK-75-mediated downregulation was very rapid, at 2 h post-treatment. Within this time period, treatment of MG132 almost completely blocked the NRF2 degradation by PIK-75 (Fig. 3B, lanes 3 vs. 4). Prolonged (4 h) PIK-75 treatment reduced the effect of MG132 (Fig. 3B, lanes 5 vs. 6). All these results suggest that PIK-75 rapidly induces NRF2 degradation by proteasome.

### PIK-75 inhibits the proliferation of pancreatic cancer cells via apoptotic cell death

**Figure 4.**
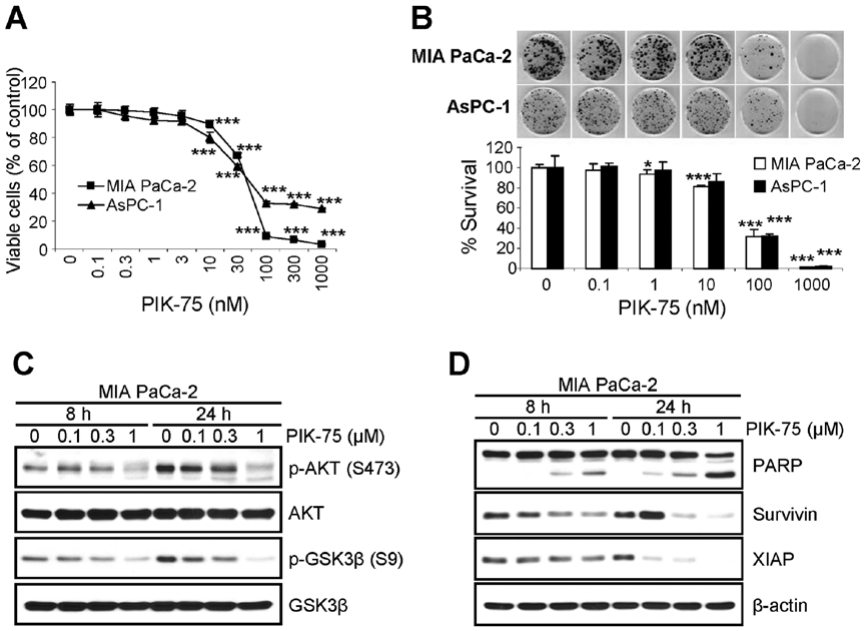
PIK-75 inhibits the proliferation and survival of pancreatic cancer cells through apoptotic cell death. (A) MIA PaCa-2 and AsPC-1 cells were treated with increasing concentration of PIK-75 for 48 h and the cell viability was determined by MTT assay. Data are presented as mean ± SD from three independent experiments performed in triplicate. (B) The cells were treated with PIK-75 as described in Materials and methods. The survival fraction was determined by crystal violet staining. Data are presented as mean ± SD from two independent experiments performed in duplicate. (A and B) ^*^P≤0.05 and ^***^P≤0.001. (C and D) MIA PaCa-2 cells were treated with PIK-75 as indicated. Western blot analysis was performed with indicated antibodies. (C) GSK3β used as a loading control. (D) β-actin was used as a loading control.

Since the depletion of NRF2 reduced the proliferation of pancreatic cancer cells (Fig. 1) and PIK-75 reduced the level of NRF2 protein in pancreatic cancer cells (Fig. 2), we tested the effect of PIK-75 on the proliferation of pancreatic cancer cells. Submicromolar concentration of PIK-75 was sufficient to inhibit the proliferation of pancreatic cancer, MIA PaCa-2 and AsPC-1 cells after 48-h treatment (Fig. 4A). The effect of PIK-75 on the survival of these pancreatic cancer cells were further evaluated by clonogenic assay. Consistently, PIK-75 also reduced the colony formation of pancreatic cancer MIA PaCa-2 and AsPC-1 cells (Fig. 4B).

The effect of PIK-75 on the PI3K/AKT signal transduction was determined in MIA PaCa-2 cells. The cells were treated with different concentrations of PIK-75 and western blot analysis was performed. As expected, PIK-75 reduced the levels of phospho-AKT (S473) and its substrate phospho-GSK3β (S9) in a dose-dependent manner within 8 h post-treatment (Fig. 4C).

To determine the markers for apoptotic cell death, MIA PaCa-2 cells were treated with increasing amount of PIK-75 and the cell lysates were subjected to western blot analysis. The PARP cleavage was evident as early as 8 h post-treatment in a dose-dependent manner (Fig. 4D). The levels of anti-apoptotic proteins including survivin and XIAP were also reduced by PIK-75 in a dose- and time-dependent manner (Fig. 4D).

### PIK-75 enhances the cytotoxicity of gemcitabine through downregulation of MRP5

**Figure 5.**
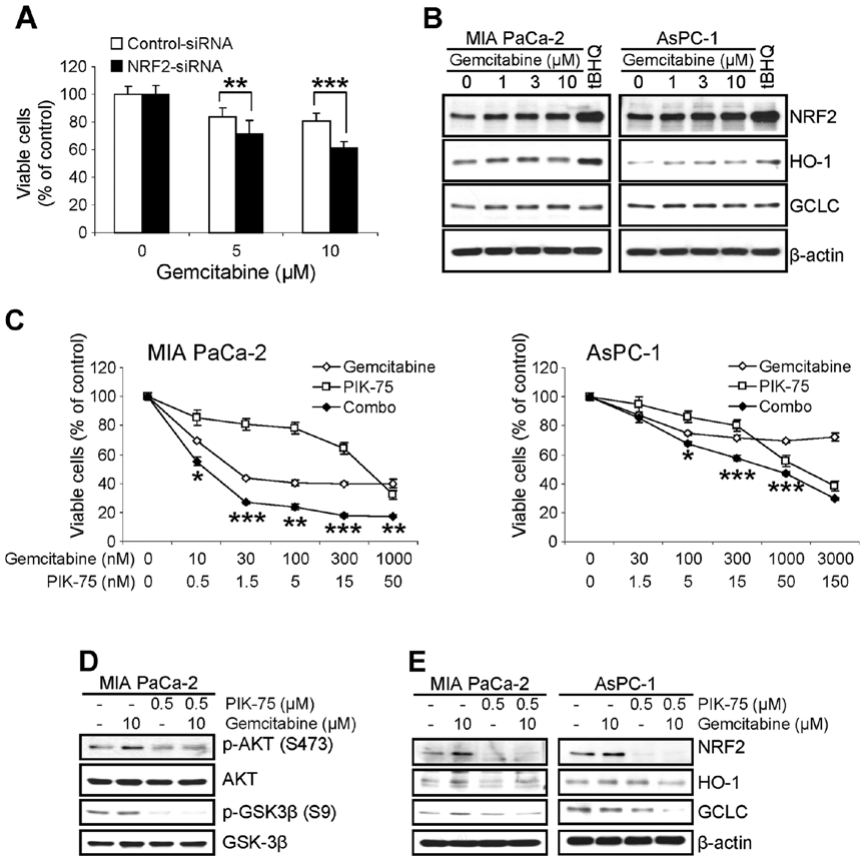
PIK-75 potentiates gemcitabine-induced cytotoxicity in pancreatic cancer cells. (A) AsPC-1 cells, transfected with siRNA (NRF2 vs. control), were treated with gemcitabine for 48 h and the viable cells were determined by MTT assay. Data are presented as mean ± SD performed in triplicate. ^**^P≤0.01 and ^***^P≤0.001. (B) MIA PaCa-2 and AsPC-1 cells were treated with increasing amount of gemcitabine for 8 h and western blot analysis was performed with indicated antibodies. β-actin was used as a loading control. (C) Cells were treated either gemcitabine, PIK-75 alone or in combination of both drugs for 48 h and viable cells were measured by MTT assay. Data are presented as mean ± SD from two independent experiments performed in triplicate. ^*^P≤0.05; ^**^P≤0.01; and ^***^P≤0.001. (D) MIA PaCa-2 cells were treated with gemcitabine, PIK-75 or combination of both drugs for 8 h and western blot analysis was performed with indicated antibodies. GSK3β used as a loading control. (E) Cells were treated with gemcitabine, PIK-75 or combination of both drugs for 8 h and western blot analysis was performed with indicated antibodies. β-actin was used as a loading control.

Since we found that NRF2 confers resistance of pancreatic cancer cells to various chemotherapeutic agents (20), we assessed the effect of NRF2-KD on the cytotoxicity of gemcitabine in pancreatic cancer cells. AsPC-1 cells were transfected with siRNA (either control or NRF2), then treated with gemcitabine for 48 h and viable cells were determined by MTT assay. Similar to other chemotherapeutic agents (20), the cytotoxic effect of gemcitabine was profound in NRF2-KD cells (Fig. 5A).

Next, we determined the effect of gemcitabine on the level of NRF2. MIA PaCa-2 and AsPC-1 cells were treated with increasing amount of gemcitabine for 8 h and the level of NRF2 and its downstream targets were determined by western blot analysis. As a control for NRF2 induction, cells were treated with 100 *μ*M of tBHQ for 8 h. Interestingly treatment of gemcitabine slightly increased the level of NRF2 in both cell lines within 8 h (Fig. 5B). The level of HO-1 protein was also slightly increased in both cells. On the contrary, slight increase of GCLC protein was only observed in MIA PaCa-2 cells (Fig. 5B).

To determine any beneficial effect by PIK-75-mediated downregulation of NRF2 in pancreatic cancer cells to cytotoxicity of gemcitabine, the effect of PIK-75/gemcitabine combination on the proliferation of pancreatic cancer cells was performed by MTT cell viability assay. The cells were treated with either gemcitabine or PIK-75 alone, or in combination of both drugs for 48 h and the viable cells were determined. As shown in Fig. 5C, co-treatment of PIK-75 profoundly enhanced the cytotoxic effect of gemcitabine in both cells with CI_50_ values of 0.1 for MIA PaCa-2 and 0.41 for AsPC-1, respectively.

The effect of PIK-75/gemcitabine combination was further analyzed by western blot analysis. Consistent with previous report (25), gemcitabine induced the phospho-AKT (S473) in MIA PaCa-2 cells after 24 h treatment (Fig. 5D). Under these conditions, PIK-75 reduced the gemcitabine-induced phospho-AKT (S473) in MIA PaCa-2 cells (Fig. 5D).

To determine the effect of PIK-75/gemcitabine combination on the NRF2 pathway, pancreatic cancer cells were treated with either drug as single agents or combination of both drugs for 8 h and western blot analysis was performed. Treatment of PIK-75 reduced the level of NRF2 and its downstream targets, HO-1 and GCLC even in the presence of gemcitabine in both cell types (Fig. 5E).

**Figure 6.**
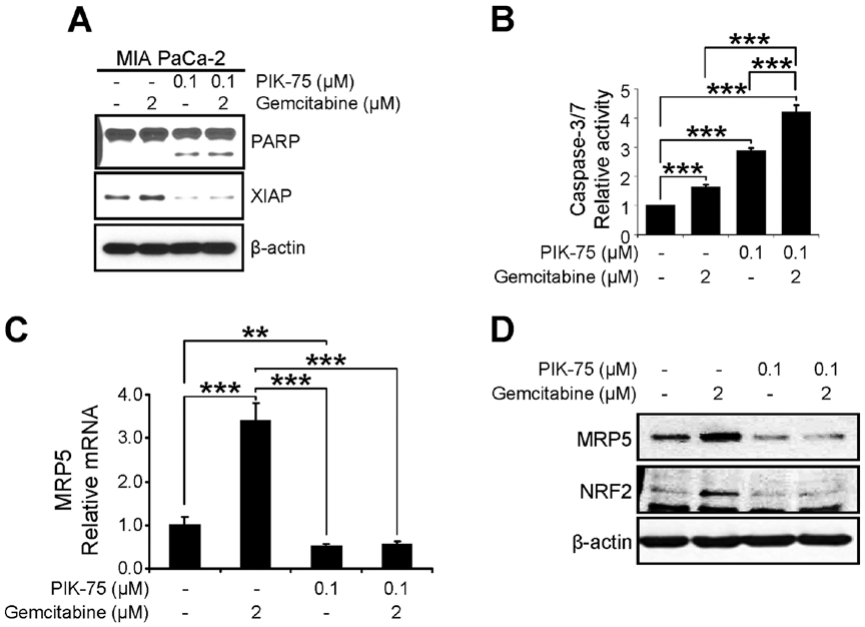
PIK-75 enhances gemcitabine-induced apoptotic cell death and reduces MRP5 expression. (A) MIA PaCa-2 cells were treated as indicated for 24 h and western blot analysis was performed with indicated antibodies. β-actin was used as a loading control. (B) MIA PaCa-2 cells were treated as described in (A) for 12 h and the caspase-3/7 activity was determined as described in Materials and methods. Data are presented as mean ± SD from two independent experiments performed in triplicate. ^***^P≤0.001. (C) MIA PaCa-2 cells were treated as indicated for 24 h and the level of MRP5 mRNA was determined by qRT-PCR. Data are presented as mean ± SD from two independent experiments performed in triplicate. ^**^P≤0.01 and ^***^P≤0.001. (D) MIA PaCa-2 cells were treated as described in (C) and western blot analysis was performed with indicated antibodies. β-actin was used as a loading control.

The anti-proliferative effect of PIK-75/gemcitabine combination was further assessed by western blot analysis of apoptotic markers. MIA PaCa-2 cells were treated with either drug or combination of both drugs for 24 h and western blot analysis was performed. As shown in Fig. 6A, PIK-75 alone induced the PARP cleavage and combination of both drugs further induced PARP cleavage. In addition, the level of the anti-apoptotic protein XIAP, was reduced by PIK-75 treatment. We further demonstrated the apoptotic cell death by measuring caspase-3/7 activity. MIA PaCa-2 cells were treated with minimal amount of gemcitabine (2 *μ*M), PIK-75 (0.1 *μ*M), or combination of both for 12 h and the caspase-3/7 activity was determined. Either gemcitabine, or PIK-75 alone induced significant activity of caspase-3/7 within 12 h (Fig. 6B). Again, PIK-75/gemcitabine combination further enhanced caspase-3/7 activity in MIA PaCa-2 cells.

The effect of PIK-75/gemcitabine on the expression of MRP5 was determined by qRT-PCR and western blot analysis. MIA PaCa-2 cells were treated with either drug as single agents or combination for 24 h. Under these conditions, gemcitabine markedly induced both mRNA and protein levels of MRP5 and co-treatment of PIK-75 reduced the gemcitabine-induced MRP5 expression (Fig. 6C and D).

### PIK-75 potentiates anticancer activity of gemcitabine in vivo

**Figure 7.**
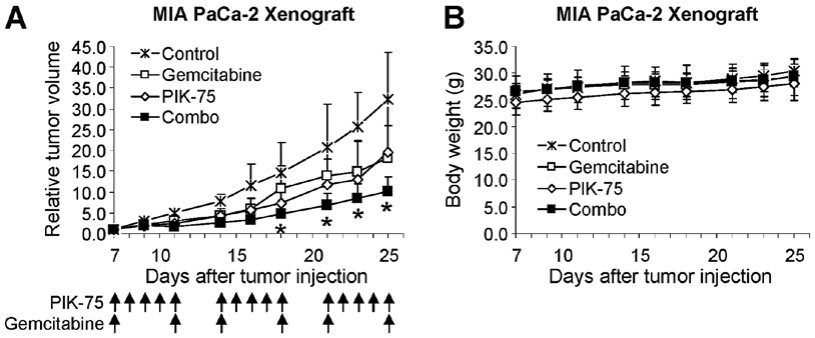
PIK-75 enhances the antitumor effect of gemcitabine *in vivo*. Mice (five mice per group) bearing tumors of MIA PaCa-2 were administered as indicated. (A) The tumor sizes were measured three times per week as described in Materials and methods. ^*^P≤0.05. (B) The body weights of mice in (A) were measured three times per week.

The effect of PIK-75/gemcitabine combination was further demonstrated by *in vivo* mouse xenograft model. Mice bearing tumors of MIA PaCa-2 were administered with gemcitabine (20 mg/kg), PIK-75 (2 mg/kg), or combination of both drugs. Since PIK-75 is a reversible inhibitor, PIK-75 was administered 5 times per week to ensure maintaining sufficient inhibitory effects. Gemcitabine was administered twice per week. As shown in Fig. 7A, gemcitabine or PIK-75 reduced the tumor growth to similar degree. Beneficial effect of PIK-75/gemcitabine was evident as this combination markedly reduced the tumor growth *in vivo* without affecting the body weights of mice (Fig. 7B).

## Discussion

In the present study, we demonstrated that PIK-75 is a potent inhibitor of NRF2 by inducing its proteasomal degradation in human pancreatic cancer cells. In addition, PIK-75 potentiated gemcitabine-induced antitumor effect through downregulation of MRP5 *in vitro*.

NRF2, by inducing expression of multiple genes that have roles in oxidative stress, detoxification, drug resistance and cell survival, functions either as a tumor protector or oncogene (27–30). In cancer cells, NRF2 induces resistance of cancer cells to chemotherapeutic drugs by upregulating transcription of various drug resistant genes such as anti-apoptotic proteins and drug transporters (12,31,32). While various activators of the NRF2 pathway have been reported to increase the level of NRF2 (33), small molecules that inhibit NRF2 activity are less defined. Since proteins that negatively regulate the NRF2 level also regulate various signaling pathways (32), it is possible that small molecule kinase inhibitors affect the level of NRF2. As an example, the MEK inhibitor AZD6244 decreased K-Ras^G12D^-induced expression of Nrf2 gene and its target genes in primary murine cells (16). As described in the present study, our initial attempt identified PIK-75 as an inhibitor of NRF2 from a small set of kinase inhibitors. Since the stability of NRF2 is regulated by PI3K/AKT/GSK3β pathway, reducing NRF2 by a PI3K/AKT pathway inhibitor can be predictable as an educated guess, in some respect. The distinct potency of PIK-75 in downregulation of NRF2, however, was unpredictable as compared to other PI3K/AKT pathway inhibitors such as PI-103. Both PIK-75 and PI-103 was reported to commonly inhibit PI3K p110α, p110δ and DNA-PK with similar degree (26). Downregulation of NRF2 by 1 *μ*M of PIK-75 was rapid at 8 h post-treatment, whereas other inhibitors required prolonged incubation time (data not shown). Further investigation will be needed to address the pathway and target responsible to this unique feature of PIK-75.

Our present study raises additional questions as to how gemcitabine induces NRF2 level in pancreatic cancer cells. Gemcitabine is known to activate various protein kinases such as ERK (34), AKT (25,34), EGFR and HER3 (34) in pancreatic cancer cells, and PKC (35) in ovarian cancer cells. Interestingly, all these kinases have implications in the regulation of NRF2 stability and/or induction: i) the MEK inhibitor AZD6244, that inhibits MEK-ERK pathway (36), reduced the K-Ras^G12D^-mediated Nrf2 induction in primary murine cells (16); ii) AKT phosphorylates GSK3β (S9) (37,38) to inhibit its kinase activity that phosphorylates NRF2 and induces its degradation by β-TrCP-dependent ubiquitination (39). GSK3β is also known to be activated by phosphorylation of Y216 by unknown upstream tyrosine kinase. Active GSK3β (Y216) phosphorylates and activates SRC family kinases that induce nuclear exclusion and ubiquitin-mediated degradation of NRF2 through its phosphorylation of Y568 (40,41); iii) activation of EGFR by EGF lead to induction of NRF2 in non-small cell lung cancer cells (18); and iv) phosphorylation of NRF2 serine 40 by PKCδ is required for stabilization and nuclear localization of NRF2 (42). Alternatively, gemcitabine induces reactive oxygen species (ROS) in pancreatic cancer cells (43,44). Increased ROS may induce stabilization/activation of NRF2 (9–12).

In the present study, induction of NRF2 by gemcitabine enhanced the expression of MRP5. It has been reported that MRP5 is a target gene of NRF2 in mouse liver by micro-array analysis (31) and knockdown of NRF2 reduced the MRP5 mRNA in pancreatic cancer cells (20,21). MRP5 is a member of MRP-related ABCC family (45). MRP5 contains two membrane-spanning domains and is known to confer resistance to cyclic nucleotides, acyclic nucleoside phosphates and monophosphorylated nucleoside analogs (46). Notably, high dose (20 *μ*M) of gemcitabine has been reported to induce expression of MRP5 mRNA and MRP5-overexpression contributed to gemcitabine resistance in HEK293 and PANC-1 cells (47). In addition, silencing MRP5 by shRNA potentiated the cytotoxicity of gemcitabine in PANC-1 pancreatic cancer cells (47). MRP5 was also reported to confer gemcitabine resistance in non-small cell lung cancer cells (48). Consistently, we found that inhibiting NRF2 by PIK-75 resulted in the reduction of MRP5 expression and potentiation of gemcitabine toxicity in pancreatic cancer cells. Importantly, this synergism showed marked reduction of *in vivo* tumor growth in a mouse xenograft model.

In conclusion, our data suggest that blocking the NRF2 pathway by small molecule inhibitors is a promising therapeutic approach to treat pancreatic cancers. While several studies suggest the potential benefit of genetic silencing of NRF2 by RNA interference to reduce proliferation and/or resistance of cancer cells to chemotherapeutics, its immediate application is hampered by inefficient delivery of nucleic acids into cells. In this aspect, small molecules are preferable for clinical applications. Notably a recent study on urethane-induced lung carcinogenesis in Nrf2−/− mouse model has also suggested NRF2 inhibitors as rational tools to prevent malignant progression of lung cancer (49). In addition, recently it has been reported that the natural compound trigonelline inhibiting NRF2 activity with unknown mechanism, enhanced antitumor effect of etoposide in mouse xenograft models of pancreatic cancers (50). Further investigations addressing more detailed mechanisms of PIK-75 in NRF2 downregulation could increase the specificity and avoid the potential side-effects of NRF2-targeting drugs.
